# Comparison of Two Surgical Techniques Based on the Semitendinosus Myocutaneous Flap in Cats

**DOI:** 10.3390/vetsci11010006

**Published:** 2023-12-20

**Authors:** Eleftheria Dermisiadou, Ioannis Panopoulos, Dimitra Psalla, Stefanos Georgiou, Aikaterini Sideri, Apostolos Galatos, Vassiliki Tsioli

**Affiliations:** 1 Clinic of Surgery, Faculty of Veterinary Science, School of Health Sciences, University of Thessaly, Trikalon 224, GR-43100 Karditsa, Greece; eldermis@vet.uth.gr (E.D.); stegeorgiou@vet.uth.gr (S.G.); ksideri@vet.uth.gr (A.S.); agalatos@vet.uth.gr (A.G.); 2 Alphavet, Veterinary Diagnostic Imaging Center, 40 Filosofon Street, GR-14564 Athens, Greece; ipanopoulos@alphavet.gr; 3 Laboratory of Pathology, Faculty of Veterinary Medicine, Aristotle University of Thessaloniki, GR-54627 Thessaloniki, Greece; dpsalla@vet.auth.gr

**Keywords:** cat, semitendinosus myocutaneous flap, split semitendinosus myocutaneous flap, skin defects

## Abstract

**Simple Summary:**

Management of distal limb defects in cats can be challenging. We assessed two myocutaneous flaps based on the semitendinosus muscle by using clinical assessment, planimetry, CT-angiography (CTA) and histological examination. In group A, we used the Semitendinosus flap (ST) based on the distal muscle pedicle whereas in group B we used the split-semitendinosus flap (SST) after longitudinal division of the muscle based on both muscle pedicles. In both groups the myocutaneous flaps were used to manage skin defects created on the medial distal tibia. The results indicate that both myocutaneous flaps offer an alternative reconstructive technique for managing distal limb skin defects in cats. Although the SST flap development was technically more demanding than the ST flap, significant clinical differences were not observed between the two techniques.

**Abstract:**

The objective of this experimental study was to compare the semitendinosus (ST) to the split-semitendinosus (SST) myocutaneous flap in covering distal limb skin defects in cats. Twenty-eight purpose-bred laboratory DSH cats were used and allocated into two groups (ST-group (A); n = 14, SST-group (B); n = 14). ST flaps, based on the distal muscle pedicle, and SST flaps, after longitudinal division of the muscle based on both muscle pedicles, were tested over skin defects created on the medial distal tibia. Clinical assessment, planimetry, CT-angiography (CTA) and histological examination were compared between groups. Days to complete flap healing between ST and SST-flaps (30.36 ± 9.1, 32.29 ± 5.44, respectively) and final total flap areas (68.36% ± 27.18, 51.83% ± 22.48, respectively) revealed no significant differences. On CTAs, the caliber of the distal caudal femoral vein on day 10 was statistically significant higher (*p* < 0.001) for group A and a significantly higher caliber of the distal caudal femoral artery on day 30 for group B (*p* = 0.021). Histology revealed statistically higher degeneration at 6 months (*p* = 0.047) for group A, and statistically higher fibrosis at 12 months (*p* = 0.019) for group B. Both ST and SST flaps had similar healing times and provided coverage of skin tibial defects in cats.

## 1. Introduction

Myocutaneous flaps offer the advantage of transferring a muscle and its overlying skin, as a unit, to an adjacent area in order to manage demanding wound defects. This flap category has a stable blood supply restored by the main vascular pedicles, as well as many direct cutaneous arteries that leave the muscle surface and nourish the skin. Myocutaneous flaps can effectively cover defects with exposed or denuded bones, can regulate blood flow more rapidly, deliver host defense mechanisms and angiogenic factors, and are able to survive in defects with obvious contamination and even when osteomyelitis is present [1,2,3,4,5,6,7,8,9,10,11,12,13].

The semitendinosus (ST) is a type III muscle according to the blood supply, supplied by two vascular pedicles, the proximal gluteal artery and vein, and the distal caudal femoral artery and vein. Each pedicle is able to support the blood flow to the entire muscle [14,15]. The use of ST as a muscle flap was reported for the treatment of external anal sphincter incompetence and also for the repair of perineal herniation (PH) in dogs and one cat [16,17]. As a myocutaneous flap technique, ST, was firstly described by Puerto and Aronson for the reconstruction of an open tibial fracture in a dog [12]. Changes in electromyography, morphology, and ultrasound in the ST of dogs after the muscle transfer for PH repair were reported in a study by Mortari [16]. The authors have recently published an article reporting the use of the ST myocutaneous flap as a reconstructive technique for the management of skin defects on the feline hindlimb, also comparing its healing time to the second intention healing of such wounds [18]. The SST as a muscle flap has been successfully used for the augmentation of the Achilles tendon repair [19] and also as a method for PH management in dogs [20].

To the authors’ knowledge, there are no reports of the SST myocutaneous flap use either in dogs or cats.

The objective of this study was to compare the use of the ST flap to the SST flap in the management of full-thickness skin defects in the distal tibia in cats. The study was designed to assess if one of the techniques would be superior or if the two techniques would be of equal effectiveness. The hypothesis evaluated was that both myocutaneous flaps would have similar efficacy as reconstructive techniques of distal limb skin defects in cats.

## 2. Materials and Methods

### 2.1. Cats

The study included 28 purpose-bred laboratory DSH cats, aged 1–4 years (18 castrated males and 10 spayed females) and of median weight 3.5 kg. Health status was based on physical examination, hematology, serum biochemistry analysis, fecal parasitology and tests for feline immunodeficiency virus and feline leukemia virus tests. The housing, handling and use of the experimental animals were performed according to the relevant national and EU legislation. The number of animals used in the study was the minimum possible, based on welfare considerations and g-power analysis. To calculate the sample size for the variable ‘days to complete flap healing’ between the ST and SST flap, a *t*-test for differences between two independent means (2 groups) was used, by giving the Power (1-β err prob) = 0.8; the effect size d = 1; and the α err prob = 0.05. The actual power of the study is 0.82.

Cats were adopted after completion of the study.

### 2.2. Study Design

In each cat, a full-thickness skin defect was created on the medial surface of the distal tibial area. Cats were randomly allocated into two groups by using a computer program (Random Number Generator). Cats in group A (n = 14) had their defects covered by an ST flap and in group B (n = 14) by an SST flap. Seven animals in each group had the ST or the SST myocutaneous flap technique performed on the right hindlimb and the rest on the left. Clinical assessment scoring and planimetric measurements were performed on days 0, 7, 14, 21 and 30 after surgery In both groups. CTA was performed on days 0, 10 and 30. Histological examination at 0 and 14 days and 6 and 12 months was performed postoperatively.

### 2.3. Presurgical Management

Cats were premedicated with dexmedetomidine (20 μg/kg, intramuscularly [IM], Dexdomitor 0.5 mg/mL; Elanco, Greece) and butorphanol (0.3 mg/kg, IM, Dolorex, 10 mg/mL; MSD, Netherlands). Anesthesia was induced with 1% propofol (2–4 mg/kg, intravenously [IV], Propofol-MCT/LCT 1%; Fresenius Kabi Hellas, Greece) and maintained with isoflurane (1∼2%, Isoflo; Abbott Laboratories, UK) in oxygen (2 L/min). A 0.9% sodium chloride solution (NS 0.9%) IV was administered at 4 mL/kg/h during anesthesia. Preoperatively, amoxycillin, clavulanic acid (20 mg/kg, subcutaneously [SC], Synulox RTU Inj. 100 mL; Zoetis, Greece) and carprofen (2 mg/kg, IV, Rimadyl; Zoetis, Greece) were administered.

### 2.4. Surgical Procedure

**Figure 1:**
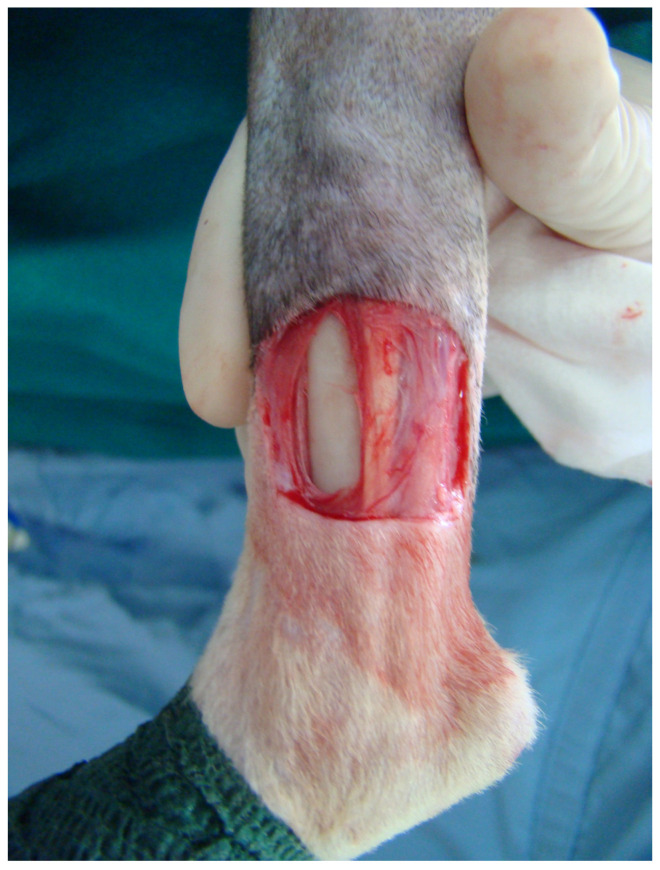
Full-thickness skin defect created on the medial surface of the distal tibial area in a cat.

A full-thickness skin defect of 2 cm in length and 1 cm in width located 1 cm above the tarsal joint was created on the medial surface of the distal tibia in each cat. The defects were approximately 40% of the circumference of the limb. The caudal tibial and medial digital flexor muscle tendons were retracted caudally. The periosteum was firstly incised and then removed, leaving the bone denuded (Figure 1).

**Figure 2:**
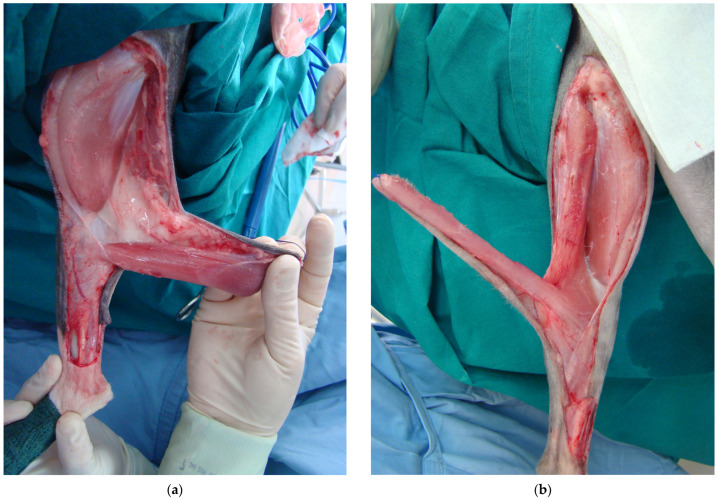
ST myocutaneous flap development in both groups: (**a**) semitendinosus myocutaneous flap elevation in group A. Note the intact distal caudal femoral pedicle entering the distal third of the semitendinosus muscle; (**b**) split-semitendinosus myocutaneous flap elevation in group B.

In group A, skin incisions extending from the ischial tuberosity to the level of the stifle joint, were created. The ST muscle was detached from its origin on the ischial tuberosity and a bridging skin incision was made between the base of the flap and the wound defect. The ST flap was rotated 150–170° distally to cover the defect (Figure 2a). In group B, the SST flap was created by splitting the muscle fibers alongside ST muscle’s longitudinal axis, starting from the muscle origin at the ischial tuberosity to the point just above the insertion of its distal pedicle. The SST flap was finally formed from the medial half of the ST muscle, which was rotated 150–170° distally to cover the defect by keeping intact the two vascular pedicles (Figure 2b). In both groups, the time between the creation of the tibial defects and the final application of the flaps ranged from 40 to 50 min.

### 2.5. Postoperative Management

Morphine hydrochloride (0.2 mg/kg IM, Morfina cloridrato; Molteni, Italy) was ad-ministered for analgesia at the end of surgery and every 4–6 h for two days and carprofen (2 mg/kg, sid, SC, Rimadyl, Zoetis, Greece) was administered for 5 days. Elizabethan collars were used until complete healing of the flaps. A 3-layer padded bandage was placed on limbs which was changed daily during the first week and then every other day until complete healing. Bandage changing and planimetric measurements were performed with the cats under sedation, which comprised dexmedetomidine (20 μg/kg, IM, Dexdomitor Elanco, Greece) and butorphanol (0.2 mg/kg, IM, Dolorex, MSD, The Netherlands).

### 2.6. Clinical Assessment Scoring

In both groups, clinical assessment was performed on days 0, 7, 14, 21 and 30 after surgery by the same person (ED) (Figures S1 and S2). A scoring system was used to quantify all the clinical observations related to the healing process of the flaps [18,21,22,23]. Pain was evaluated by using the Colorado State University Feline Acute Pain Scale [24,25] while cats were alert, once before and then after the bandage changing. Healing scores were calculated according to the criteria described in recent reports [18].

### 2.7. Planimetry

Digital images of the flaps and a computer software (AutoCAD, version 2018, Autodesk, San Rafael, CA, USA) were used for planimetry evaluation. Planimetry was used in order to measure flaps’ length, width (cm), the initial total flap area (cm^2^) (skin flap surface on day 0), the final total flap area (cm^2^) (percentage of skin flap area viable on day 30) and the percentage of flap necrosis on days 7, 14, 21 and 30, according to previous reports [18,26,27,28].

### 2.8. CT-Angiography

All flaps were studied by Computed Tomography and pre- and post-intravenous contrast medium administration (iobitridol (2 mL/kg, IV, Xenetix^®^ inj. Sol. 300 mg/mL; Guerbet, France)), which was was performed to evaluate the arterial and venous phase. The same anesthetic protocol described in Presurgical Management was used during the CTA.

**Figure 3:**
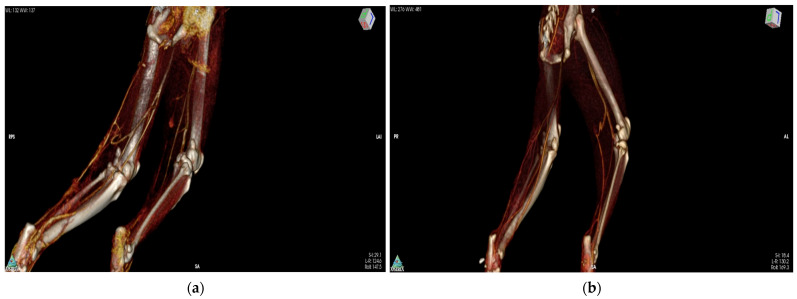
Neovascularization of the myocutaneous flaps and vascular anastomoses at the donor site on the 3-dimensional computed tomography-angiography images 10 days postoperatively (**a**) in group A (SΤ flap) and; (**b**) in group B (SST flap).

The following parameters were evaluated: ST muscle length and width (cm), the caliber of the proximal gluteal and distal caudal femoral artery and vein (mm) and the density of ST muscle (Hounsfield unit [HU] at 10 mm^2^) at three different points (dP: proximal, dM: medial, and dD: distal) in three different phases (pre-contrast agent, arterial and venous phase) [18]. [Figure 3 and supplementary figures (Figures S3 and S4)].

### 2.9. Histological Evaluation of the ST and SST Myocutaneous Flaps

On days 0 and 14, tissue samples were collected by using a 6 mm biopsy punch (Biopsy punch; Kruuse, Langeskov, Denmark). For day 0 sampling, the distal tip of each flap was chosen, whereas on day 14 specimens were collected from the medial part of the distal edge of each flap, preserving the flaps’ microcirculation [18]. Specimens of 1 cm in width by 2 cm in length were collected from the middle of the medial part of all flaps, including only the muscle parenchyma, on 6- and 12 months.

All samples were processed using standard techniques and stained with H&E. The sections were evaluated to assess inflammation, degeneration, necrosis, neovascularization, fibrosis and edema, quantitatively classified as: 0 (Normal): no alterations present, 1 (+): alterations of up to 33%, 2 (++): alterations of between 34 and 66% and 3 (+++): alterations of between 67 and 100% [18,29,30,31].

### 2.10. Statistical Analysis

Continuous data are presented as mean ± SD (e.g., days to complete flap healing, CTA-measurements), while ordinal data are presented as median and range (R) (clinical assessment score, histological score). Qualitative data are presented as frequency and percentages.

The Shapiro–Wilk test was used to evaluate the normality of continuous data. To examine differences of the continuous data following normal distribution, we employed one-way ANOVA with repeated measures and Paired *t*-test for tests within the same group, and *t*-test for comparisons between the two groups. To evaluate differences of ordinal data and continuous data rejecting the hypothesis of normality, Friedman and Wilcoxon Signed Rank test (comparisons in pairs) were performed for tests within the same group, and Mann–Whitney U test was used to examine differences between the two groups.

The Pearson correlation coefficient was used to evaluate the linear relationship between two continuous variables.

SPSS Statistical software was used in this study in order to perform all the tests needed (SPSS, version 28.0, IBM-SPSS Science, Chicago, IL, USA). Statistical significance was defined as *p* < 0.05.

## 3. Results

### 3.1. Clinical Assessment Scoring

The mean time to complete flap healing between group A (30.36 days, range: 20–52) and group B (32.29 days, range: 23–40) had no significant difference. Necrosis of the overlying skin located at the tip of the flaps was observed in 11 cats in group A (78.6%) and in 12 cats in group B (85.7%).

Clinical assessment scores in groups A and B are shown in Table 1. Statistically significant differences were observed in group A between days 7 and 30 (*p* = 0.027), 14 and 30 (*p* = 0.002), and 21 and 30 (*p* = 0.012). In group B, statistically significant differences were observed between days 7 and 14 (*p* = 0.024), 21 and 30 (*p* = 0.011) and 14 and 30 (*p* = 0.022).

Group B showed statistically significantly higher assessment scores on day 7 compared to group A (*p* = 0.031).

All cats had a pain score bellow 2 using the Colorado State University Feline Acute Pain Scale.

### 3.2. Planimetry

**Figure 4:**
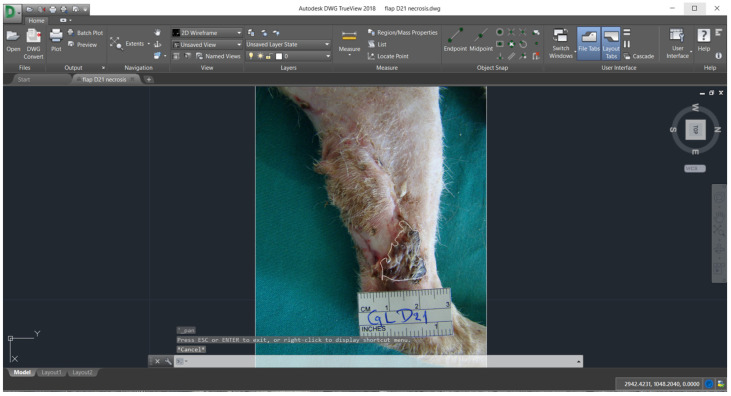
Measurement of the area with skin necrosis in a cat from group A (day 21).

**Figure 5:**
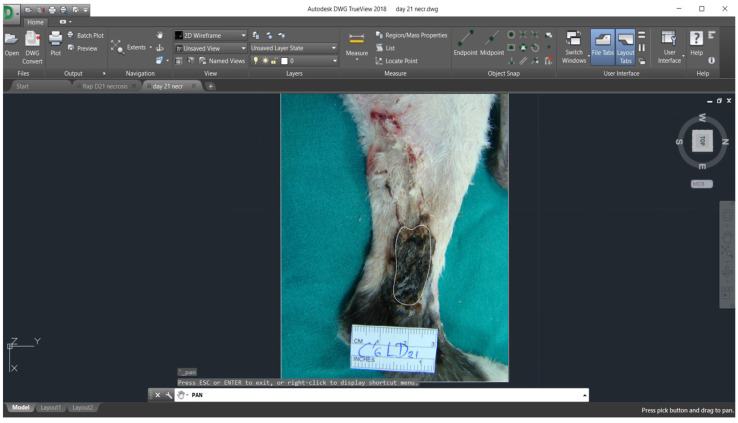
Measurement of the area with skin necrosis in a cat from group B (day 21).

Planimetric measurements in groups A and B are shown in Table 2 (Figure 4 and Figure 5). In group A, statistically significant difference was observed in % of flap necrosis, which was higher on day 14 compared to 7 (*p* = 0.013). In group B, statistically significant differences were observed in % of flap necrosis, which was higher on day 14 compared to 7 and on day 21 compared to 30 (*p* = 0.016 and *p* = 0.043, respectively). A significant positive correlation was found in group B between flap assessment score and neovascularization (histological parameter) on day 14 (*p* = 0.014).

In group B, the % of flap necrosis measurements was found to be higher than those in group A; however, no statistically significant differences were observed between the two groups. No statistically significant differences between the two groups were observed on the initial total flap area and on % of final flap area.

### 3.3. CT-Angiography

CTA measurements in groups A and B are shown in Table 3. Ιn group A, significant differences were observed in muscle width, which was higher on day 10 compared to 0 (*p* < 0.001), on day 30 compared to 0 (*p* = 0.009), and on day 10 compared to 30 (*p* = 0.008). Significantly higher measurements of ST muscle density were observed on day 10 compared to that on day 0 in all three different phases of the CTA: (a) for the proximal (dP) point (*p* < 0.001), (b) for the medial (dM) point (*p* = 003, *p* < 0.001, *p* < 0.001, respectively) and (c) for the distal (dD) point (*p* = 0.014, *p* = 0.037, *p* = 0.07, respectively). Significantly higher measurements of ST muscle density were observed on day 10 compared to day 30 in all three different phases of the CTA: (a) for the proximal (dP) point (*p* = 0.001, *p* < 0.001, *p* < 0.001, respectively), (b) for the medial (dM) point (*p* = 017, *p* < 0.001, *p* < 0.001, respectively) and (c) for the distal (dD) point (*p* = 0.024, *p* < 0.001, *p* < 0.001, respectively). Significant differences were observed in the caliber of the distal caudal femoral artery and vein, which was higher on day 10 compared to 0 and on day 10 compared to 30 (*p* < 0.001).

In group B, significant differences were observed in ST muscle density, which was higher on day 10 compared to 0 in all three different phases of the CTA: (a) in all three different phases of the CTA for the proximal point (dP) (*p* < 0.001), (b) for the medial point (dM) in the arterial and the venous phase (*p* < 0.001) and (c) for the distal point (dD), the measurements were found significantly higher on day 10 only in the venous phase (*p* = 0.028). Significant differences were also observed in ST muscle density which was higher on day 10 compared to 30 (*p* ≤ 0.001) on all points in all three different phases. Significant differences were observed in the caliber of both vascular pedicles, which were higher on day 10 compared to 0 (*p* < 0.001) and on day 10 compared to 30 (*p* ≤ 0.001). As for the comparison between day 30 and 0, the proximal gluteal vein (*p* = 0.001) and the distal caudal femoral artery (*p* = 0.014) presented significantly higher calibers on day 30.

No statistically significant differences were observed in muscle length and width between the two groups. Group A showed a statistically significant higher caliber of the distal caudal femoral vein on day 10 (*p* < 0.001) compared to group B. Group B a showed statistically significant higher caliber of the distal caudal femoral artery on day 30 (*p* = 0.021) compared to group A. No significant differences were observed in muscle density between the two groups on all points in all three different phases. No significant correlations were observed between planimetric, CTA and histological parameters between the two groups.

### 3.4. Histological Evaluation of the ST and SST Myocutaneous Flaps

The results of histological evaluation of the various parameters examined in the tissue samples are shown in Table 4. In group A, significant differences were observed on inflammation, muscle cell degeneration, neovascularization and edema, which were higher on day 14 compared to 0 (*p* = 0.001, *p* = 0.026, *p* = 0.015, *p* = 0.005, respectively) and 6 months (*p* = 0.020, *p* = 0.047, *p* = 0.05, *p* = 0.038, respectively). Fibrosis was scored as significantly higher on day 14 compared to day 0 (*p* = 0.005). No statistically significant differences in any of the parameters were observed between the time interval of 6 and 12 months. No significant correlation was observed between flap assessment scores and histological parameters on day 14.

In group B, significant differences were observed in inflammation, muscle cell degeneration and neovascularization, which were higher on day 14 compared to 0 (*p* = 0.001, *p* = 0.002, *p* = 0.005, respectively) and 6 months (*p* = 0.016, *p* = 0.014, *p* = 0.046, respectively). Fibrosis was scored as significantly higher on day 14 compared to day 0 (*p* = 0.024). No statistically significant differences in any of the parameters were observed between the time interval of 6 and 12 months. No significant correlation was observed between flap assessment scores and histological parameters on day 14.

**Figure 6:**
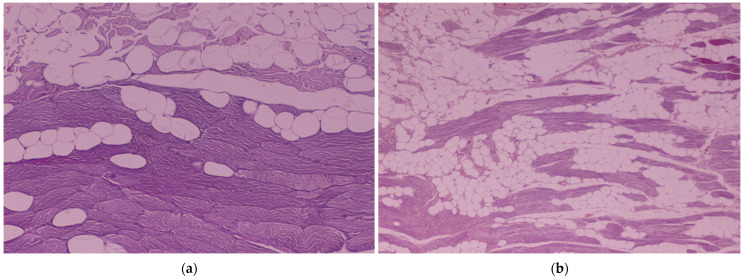
Histological images of the semitendinosus muscle: (**a**) a sample with fatty degeneration, 6 months postoperatively (haematoxylin and eosin stain, ×100 magnification); (**b**) multifocal fatty degeneration and atrophy of myofibers in a sample on the 12 months evaluation (haematoxylin and eosin stain, ×40 magnification).

Comparing the results of the two groups, significant differences were observed in degeneration at 6 months (*p* = 0.047), which was statistically higher in group A, and on fibrosis at 12 months examination (*p* = 0.019), which was significantly higher in group B compared to A (Figure 6). No statistically significant differences were observed between the two groups in the rest parameters on the other time intervals.

## 4. Discussion

The ST and SST muscle flaps are methods that have already been described in the veterinary literature [14,15,16,17,19,20]. The ST as a myocutaneous flap has been reported as an effective reconstructive technique in only one dog with a severe open tibial fracture [12]. The ST myocutaneous flap and its use for distal tibial defects in cats was recently reported by the authors of [18]. To the authors’ knowledge, this is the first study investigating the outcomes of the use of the ST and the SST myocutaneous flaps as reconstructive techniques in cats. The results of the current study suggest that both methods offer an alternative for covering full-thickness skin defects on the distal limbs in cats. Based on these findings, both myocutaneous flaps had similar healing times and did not affect the motor function of the hindlimbs.

The blood supply of the distal caudal femoral artery alone was confirmed to be sufficient for the entire ST flap survival [14,15,18]. For the SST flap formation, the two vascular pedicles of the muscle were preserved and efficiently nourished the myocutaneous flap [20].

SST flap development was technically more demanding than the ST flap only at the point of the longitudinal muscle dissection in order to preserve both the two vascular pedicles of the muscle. Both ST and SST flaps provided an ample coverage of the defects created on the tibia. The flaps were long enough to cover the defects of the distal part of the tibia without restricting their blood supply [12,18]. Although shearing forces at the distal tip of the flaps were present in both groups [18], this phenomenon most frequently appeared on the narrower myocutaneous flaps of group B and consisted of the medial half of the ST muscle [32]. These forces may detach the skin from the fascial surface of the muscle and might result in vascular compromise or even complete disruption of the skin blood flow topically. The higher clinical assessment scores in group B compared to group A on day 7 are probably attributed to these shearing forces in combination with microvascular compromise due to muscle fiber splitting [32].

The skin in both flap groups appeared initially erythematic and then congested for the first 5 days. This might be due to inconstant blood flow and insufficient venous drainage. After day 6 skin recovered or became necrotic [33,34]. The skin necrosis observed at the distal tip of some of the flaps (Figures S1 and S2) in both groups was attributed to the insufficient perfusion of the skin or to a disruption in the microvessels, such as thrombosis of arterioles or venules, reperfusion injury or regional injury of the cutaneous circulation by shearing forces [18,33,34,35,36]. These necrotic skin lesions deteriorated from day 7 to 14 but improved from day 14 to 21 in both groups [18]. On day 30, the skin appearance in group B was improved, as the % of flap necrosis was significantly lower compared to day 21. As already mentioned, in group B the hemodynamic changes were set earlier (on day 7) in the skin flap microcirculation and this might have led to a faster recovery (Figure S1). On day 30, no statistically significant difference was found between groups concerning the mean viable final total skin area, which was 68.36% for group A and 51.83% for group B.

The time to complete flap healing was similar for both groups and this is attributed to the stable blood supply provided by the pedicles in both flap techniques. Skin necrosis was observed in 11 cats in group A (78.6%) and in 12 cats in group B (85.7%), and healed by second intention. Open-wound management was performed by daily flushing with Lactated Ringer’s solution and bandaging. Complete healing was achieved without the need for further surgery. The ST muscle underneath the skin surface of the flap remained viable in all cases in both groups (100%). Serious complications, such as partial or complete flap loss, were not noticed. Despite the potential complications, myocutaneous flaps are advantageous in injuries of the extremities where there is limited tissue availability [19]. Especially in complex wounds of the distal limbs with bone injury and extensive skin loss the use of these autologous, composite flaps improve vascularity, decrease the rate of contamination, promote bone healing and oxygenates tissues [6,12,15,37]. In such cases of large complex wounds, second intention healing might result from delayed healing or nonhealing of the defect [12,14]. Moreover, second intention healing might warrant prolonged hospitalization and nursing care, as well as a lot of bandaging products and medications, which must be weighed out. On the other hand, another option would be to initially use the semitendinosus as a muscle flap and afterwards to cover the transposed muscle with a skin graft or, in cases of defects without extreme skin loss, to transpose the muscle and let skin heal by second intention [12]. Further studies are needed to evaluate the use of the ST muscle flap combined with either a graft or by second intention healing to avoid distal skin necrosis. Microvascular free-tissue transfer also offers the advantages of covering a defect with various tissues in a contaminated environment and of early reconstruction of complex wounds in one surgical procedure. However, in distal limb defects with extensive vascular damage its use may be limited. Moreover, it requires special training and instrumentation [14,38]. CTA on day 10 confirmed that there was an undisturbed blood flow to the flaps in both techniques, and no trauma, kinking, rotation or thrombus formation of the preserved pedicles was present. In both groups an increased muscle density was present and larger vessel caliber measurements were recorded on day 10 compared to 0. This indicates that incremental vasodilatation, neovascularization and new choke anastomoses established adequate blood flow to the flap [6]. In group A, the significantly larger caliber of the main pedicle on day 10 compared to day 30 and the absence of differences between day 0 and 30 indicate that the vessel’s caliber returned to its initial size by day 30 [15,18]. In group B there was a significantly larger caliber of both pedicles on day 10 compared to day 30 and a significantly larger diameter of the proximal gluteal vein and the distal caudal femoral artery on day 30 compared to 0. This probably reveals that the blood flow requirements in group B were higher in order to meet the needs of both the transferred flap and the half of the semitendinosus muscle which remained in place.

The histological examination revealed that the ST and SST flaps did not present major muscle necrosis during the whole procedure [6,15,18,30]. In both flap techniques a stable blood supply was preserved, and it seems that both vascular pedicles can satisfactorily manage the nourishing requirements of the myocutaneous flaps. On day 0, necrosis and edema were observed in some specimens of both groups. These findings are probably associated with the tissue handling and the use of the electrocautery. On day 14, significantly higher scores were found in all parameters compared to day 0, due to the inflammatory response in the wound healing process, the possible limited blood flow and the reperfusion injury that was observed in both groups postoperatively at the sampling points [18,28,29,30,31]. Histological examination of the samples on 6 and 12 months was characterized by muscle cell degeneration, probably due to muscle cell replacement by fatty and fibrous tissue after the neurovascular division [18,30]. Higher degeneration scores were found in histological examination in group A compared to B at 6 months. Preservation of both nerve pedicles in group B could possibly be responsible for this delay in the muscle degenerative process at that time period compared to group A. Higher muscle fibrosis in group B compared to A was evident at all time points, although it was significant higher only on the 12 months examination. This could possibly be attributed to the trauma that the flaps underwent during the surgical muscle split in group B.

The study limitations include the small number of animals because of ethic and welfare concerns. Evaluation of the wound healing and scoring could not be performed blindly. Further investigation of skin flap perfusion with the use specified evaluating methods (laser Doppler flowmetry, fluorescein dye) are needed for both myocutaneous flaps depending on the ST muscle [39,40,41,42]. The management of clean surgical wounds, such as reported in this study, may not highlight the significance of myocutaneous flap development; however, the use of the ST and the SST flaps in clinical cases may offer new advantageous techniques to manage complex distal limb injuries in cats. Finally, the area that can be covered within the arc of rotation of these flaps was not studied and it would be interesting to define it in the future.

In conclusion ST and SST flaps should be considered as alternative techniques for the reconstruction of full-thickness skin wounds in the distal tibial area in cats.

## Figures and Tables

**Table 1:** Clinical assessment scores in groups A and B. Values are presented as median and range (R).

Clinical Assessment Score	Day 7	Day 14	Day 21	Day 30
Group A	8.5 (3–10)	7.5 (0–27)	7 (0–22)	0 (0–19)
Group B	10 (7–15)	12.5 (8–18)	11.5 (0–20)	7 (0–18)

**Table 2:** Planimetric measurements in groups A and B. Values are presented as mean ± standard deviation (SD).

Planimetric Measurements	Group A	Group B
Flap width (cm)	1.64 ± 0.30	1.49 ± 0.23
Flap length (cm)	7.27 ± 0.46	6.95 ± 0.42
Initial total flap area (cm^2^)	11.79 ± 1.44	9.06 ± 1.47
% final total flap area	68.36 ± 27.18	51.83 ± 22.48
% flap necrosis on day 7	12.39 ± 27.03	12.13 ± 22.39
% flap necrosis on day 14	28 ± 26	34.83 ± 17.23
% flap necrosis on day 21	21.07 ± 28.23	16.57 ± 24.04
% flap necrosis on day 30	8.52 ± 16.33	0.63 ± 2.34

**Table 3:** Parameters studied on CT-angiographies in groups A and B. Values are presented as mean ± standard deviation (SD).

Parameters		Day 0	Day10	Day 30
Muscle length (cm)	group A	10.76 ± 0.97	NA	NA
	group B	11.03 ± 0.3	NA	NA
Muscle width day 0 (cm)	group A	2.11 ± 0.19	2.25 ± 0.26	2.16 ± 0.2
	group B	1.97 ± 0.11	NA	NA
Proximal gluteal artery caliber (mm)	group A	0.85 ± 0.02	NA	NA
	group B	0.84 ± 0.02	1.02 ± 0.05	0.86 ± 0.02
Proximal gluteal vein caliber (mm)	group A	1.25 ± 0.05	NA	NA
	group B	1.25 ± 0.02	1.41 ± 0.13	1.28 ± 0.02
Distal caudal femoral artery caliber (mm)	group A	0.86 ± 0.05	1.03 ± 0.04	0.86 ± 0.03
	group B	0.86 ± 0.02	1.02 ± 0.04	0.89 ± 0.04
Distal caudal femoral vein caliber (mm)	group A	1.26 ± 0.03	1.50 ± 0.04	1.26 ± 0.19
	group B	1.25 ± 0.02	1.40 ± 0.05	1.27 ± 0.02
Muscle density-proximal point when no contrast agent given (dP) (HU)	group A	52.49 ± 3.73	57.89 ± 3.3	55.98 ± 3.24
	group B	52.38 ± 2.34	56.00 ± 2.54	54.46 ± 2.47
Muscle density-medial point when no contrast agent given (dM) (HU)	group A	54.05 ± 3.72	55.02 ± 3.3	54.39 ± 3.26
	group B	54.35 ± 1.85	55.05 ± 2.16	53.36 ± 2.17
Muscle density-distal point when no contrast agent given (dD) (HU)	group A	52.94 ± 3.71	54.17 ± 3.16	51.85 ± 3.77
	group B	53.16 ± 2.45	53.45 ± 2.2	52.13 ± 1.99
Muscle density on the proximal point—arterial phase (dPa) (HU)	group A	67.49 ± 5.52	87.63 ± 4.93	81.87 ± 3.28
	group B	65.08 ± 3.65	85.39 ± 4.09	80.46 ± 2.72
Muscle density on the medial point—arterial phase (dMa) (HU)	group A	71.92 ± 5.05	79.50 ± 2.70	74.31 ± 2.6
	group B	69.52 ± 3.87	79.07 ± 2.03	74.67 ± 1.94
Muscle density on the distal point—arterial phase (dDa) (HU)	group A	69.3 ± 5.36	71.88 ± 1.76	67.87 ± 2.12
	group B	66.98 ± 3.54	66.77 ± 8.82	63.42 ± 8.15
Muscle density on the proximal point—venous phase (dPv) (HU)	group A	73.65 ± 5.47	93.88 ± 5.7	87.40 ± 3.59
	group B	71.20 ± 4.59	92.69 ± 4.83	86.89 ± 2.88
Muscle density on the medial point—venous phase (dMv) (HU)	group A	78.21 ± 6.73	84.25 ± 2.92	78.11 ± 2.53
	group B	74.19 ± 3.44	86.50 ± 4.12	79.42 ± 2.18
Muscle density on the distal point—venous phase (dDv) (HU)	group A	75.23 ± 5.15	78.92 ± 2.43	73.22 ± 1.47
	group B	72.62 ± 4	76.52 ± 4.38	70.63 ± 5.83

Abbreviation: NA, not applicable.

**Table 4:** Results of the various parameters studied during histological evaluation of tissue samples in groups A and B presented as median values and range (min–max).

Histological Parameters		Day 0 (n = 12)	Day 14 (n = 12)	6 Months (n = 6)	12 Months (n = 6)
Inflammation	group A	0 (0)	2 (0–2)	0 (0–1)	0 (0–2)
	group B	0 (0)	2 (0–3)	0 (0–1)	0 (0–2)
Degeneration/Necrosis	group A	2 (1–2)	3 (0–3)	2 (1–2)	1 (0–3)
	group B	2 (1–3)	3 (1–3)	1 (0–2)	1 (0–2)
Neovascularization	group A	0 (0)	0,5 (0–2)	0 (0)	0 (0–1)
	group B	0 (0)	1 (0–2)	0 (0)	0 (0–1)
Fibrosis	group A	0 (0)	1 (0–3)	0 (0–1)	0 (0–1)
	group B	0 (0)	0 (0–3)	1 (0–1)	1 (0–1)
Edema	group A	1 (0–1)	1 (1–2)	0 (0–1)	1 (0–2)
	group B	1 (0–1)	1 (0–3)	0 (0–1)	1 (0–1)

## Data Availability

The data presented in this study are available in the article.

## Supplementary Materials

The following supporting information can be downloaded at: https://www.mdpi.com/article/10.3390/vetsci11010006/s1, Figure S1: The healing process of a ST flap (group A); Figure S2: The healing process of a SST flap (group B); Figure S3: Evaluation of the density of the semitendinosus muscle (HU at 10 mm2) in group A; Figure S4: Evaluation of the density of the semitendinosus muscle (HU at 10 mm2) in group B.

## References

[1] McCraw J.B., Dibbell D.G., Carraway J.H. (1977). Clinical definition of independent myocutaneous vascular territories. Plast. Reconstr. Surg..

[2] Ariyan S., Cuono C.B. (1980). Myocutaneous Flaps for Head and Neck Reconstruction. Head Neck Surg..

[3] Mathes S.J., Nahai F. (1981). Classification of the vascular anatomy of muscles: Experimental and clinical correlation. Plast. Reconstr. Surg..

[4] Pavletic M.M., Kostolich M., Koblik P., Engler S. (1987). A comparison of the cutaneous trunci myocutaneous flap and latissimus dorsi myocutaneous flap in the dog. Vet. Surg..

[5] Taylor G.I., Palmer J.H. (1987). The vascular territories (angiosomes) of the body—Experimental study and clinical applications. Br. J. Plast. Surg..

[6] Pavletic M.M. (1990). Introduction to myocutaneous and muscle flaps. Vet. Clin. N. Am. Small Anim. Pract..

[7] Taylor G.I., Minabe T. (1992). The angiosomes of the mammals and other vertebrates. Plast. Reconstr. Surg..

[8] De Mello-Filho F.V., Mamede R.C., Sader A.A., Velludo M.A., Vicente W.V. (1993). Use of the platysma myocutaneous flap for cervical trachea reconstruction: An experimental study in dogs. Laryngoscope.

[9] Smith Μ., Shults S., Waldron D.R., Moon M.L. (1993). Platysma myocutaneous flap for head and neck reconstruction in cats. Head Neck.

[10] Degner D.A., Walshaw R., Arnoczky S.P., Smith R.J., Patterson J.S., Degner L.A., Hamaide A., Rosenstein D. (1996). Evaluation of the cranial rectus abdominus muscle pedicle flap as a blood supply for the caudal superficial epigastric skin flap in dogs. Vet. Surg..

[11] Taylor G.I. (2003). The angiosomes of the body and their supply to perforator flaps. Clin. Plast. Surg..

[12] Puerto D.A., Aronson L.R. (2004). Use of a semitendinosus myocutaneous flap for soft-tissue reconstruction of a grade IIIB open tibial fracture in a dog. Vet. Surg..

[13] Doyle C.P., Degner D.A. (2019). Evaluation of the Superior Labial Musculomucosal Flap in Dogs: An Angiographic Study and Case Report. Vet. Comp. Orthop. Traumatol..

[14] Chambers J.N., Rawlings C.A. (1991). Applications of a semitendinosus muscle flap in two dogs. J. Am. Vet. Med. Assoc..

[15] Solano M., Purinton P.T., Chambers J.N., Munnell J.F. (1995). Effects of vascular pedicle ligation on blood flow in canine semitendinosus muscle. Am. J. Vet. Res..

[16] Mortari A.C., Rahal S.C., Resende L.A., Dal-pai-silva M., Mamprim M.J., Corrêa M.A., Antunes S.H. (2005). Electromyographical, ultrasonographical and morphological modifications in semitendinous muscle after transposition as ventral perineal muscle flap. J. Vet. Med. A Physiol. Pathol. Clin. Med..

[17] Vnuk D., Babić T., Stejskal M., Capak D., Pirkić B., Harapin I. (2005). Application of a semitendinosus muscle flap in the treatment of perineal hernia in a cat. Vet. Rec..

[18] Dermisiadou E., Panopoulos I., Psalla D., Georgiou S., Sideri A., Galatos A., Tsioli V. (2023). Use of a semitendinosus myocutaneous flap for the coverage of hindlimb full-thickness skin defects in cats. J. Vet. Sci..

[19] Baltzer W.I., Rist P. (2009). Achilles tendon repair in dogs using the semitendinosus muscle: Surgical technique and short-term outcome in five dogs. Vet. Surg..

[20] Morello E., Martano M., Zabarino S., Piras L.A., Nicoli S., Bussadori R., Buracco P. (2015). Modified semitendinosus muscle transposition to repair ventral perineal hernia in 14 dogs. J. Small Anim. Pract..

[21] Lazarus G.S., Cooper D.M., Knighton D.R., Margolis D.J., Percoraro R.E., Rodeheaver G., Robson M.C. (1994). Definitions and guidelines for assessment of wounds and evaluation of healing. Arch. Dermatol..

[22] Grey J.E., Enoch S., Harding K.G. (2006). Wound assessment. BMJ.

[23] Ousey K., Cook L. (2012). Wound Assessment: Made Easy. Wounds.

[24] Epstein M.E., Rodan I., Griffenhagen G., Kadrlik J., Petty M., Robertson S., Simpson W. (2015). 2015 AAHA/AAFP pain management guidelines for dogs and cats. J. Feline Med. Surg..

[25] Mathews K., Kronen P.W., Lascelles D., Nolan A., Robertson S., Steagall P.V., Wright B., Yamashita K. (2014). Guidelines for recognition, assessment and treatment of pain: WSAVA Global Pain Council members and co-authors of this document. J. Small Anim. Pract..

[26] Bohling M.W., Henderson R.A., Swaim S.F., Kincaid S.A., Wright J.C. (2004). Cutaneous wound healing in the cat: A macroscopic description and comparison with cutaneous wound healing in the dog. Vet. Surg..

[27] Bohling M.W., Henderson R.A. (2006). Differences in cutaneous wound healing between dogs and cats. Vet. Clin. N. Am. Small Anim. Pract..

[28] Bohling M.W., Henderson R.A., Swaim S.F., Kincaid S.A., Wright J.C. (2006). Comparison of the role of the subcutaneous tissues in cutaneous wound healing in the dog and cat. Vet. Surg..

[29] Costa W., da Silva A.L., Costa G.R., Nunes T.A. (2012). Histology of the rectus abdominis muscle in rats subjected to cranial and caudal devascularization. Acta Cirúrgica Bras..

[30] Greaves P., Chouinard L., Ernst H., Mecklenburg L., Pruimboom-Brees I.M., Rinke M., Rittinghausen S., Thibault S., Von Erichsen J., Yoshida T. (2013). Proliferative and Non-Proliferative Lesions of the Rat and Mouse Soft Tissue, Skeletal Muscle and Mesothelium. J. Toxicol. Pathol..

[31] Siemionow M., Manikowski W., Gawronski M. (1995). Histopathology of muscle flap microcirculation following prolonged ischemia. Microsurgery.

[32] Pavletic M.M. (1980). Vascular supply to the skin of the dog: A review. Vet. Surg..

[33] Pavletic M.M. (1990). Axial pattern flaps in small animal practice. Vet. Clin. N. Am. Small Anim. Pract..

[34] Pavletic M.M. (1981). Canine axial pattern flaps, using the omocervical, thoracodorsal, and deep circumflex iliac direct cutaneous arteries. Am. J. Vet. Res..

[35] Pang C.Y. (1990). Ischemia-induced reperfusion injury in muscle flaps: Pathogenesis and major source of free radicals. J. Reconstr. Microsurg..

[36] Kerrigan C.L., Stotland M.A. (1993). Ischemia reperfusion injury: A review. Microsurgery.

[37] Spadola F., Neve V.C., Interlandi C.D., Spadaro A., Macrì F., Iannelli N.M., Costa G.L. (2022). Hernioplasty with Peritoneal Flap for the Surgical Treatment of Umbilical Hernia in Swine. Animals.

[38] Jackson A.H., Degner D.A., Jackson I.T., Miyawaki T., Silverberg B., Bradford M., Andrus L. (2003). Deep circumflex iliac cutaneous free flap in cats. Vet. Surg..

[39] Lanthier T., Miller C., McDonell W.N., Yager J.A., Roth J.H. (1990). Use of laser Doppler flowmetry to determine blood flow in and viability of island axial pattern skin flaps in rabbits. Am. J. Vet. Res..

[40] Pratt G.F., Rozen W.M., Chubb D., Whitaker I.S., Grinsell D., Ashton M.W., Acosta R. (2010). Modern adjuncts and technologies in microsurgery: An historical and evidence-based review. Microsurgery.

[41] Losken A., Styblo T.M., Schaefer T.G., Carlson G.W. (2008). The use of fluorescein dye as a predictor of mastectomy skin flap viability following autologous tissue reconstruction. Ann. Plast. Surg..

[42] Lee J.H., You H.J., Lee T.Y., Kang H.J. (2022). Current Status of Experimental Animal Skin Flap Models: Ischemic Preconditioning and Molecular Factors. Int. J. Mol. Sci..

