# Supplementary material for: Comparison of Two Surgical Techniques Based on the Semitendinosus Myocutaneous Flap in Cats
Source: Vet Sci. 2023 Dec 20;11(1):6. doi: 10.3390/vetsci11010006 (PMC10818443; doi:10.3390/vetsci11010006)
Supplement: Supplementary file 1 [file vetsci-11-00006-s001.zip › vetsci-2709702-supplementary.pdf]

Supplementary Materials

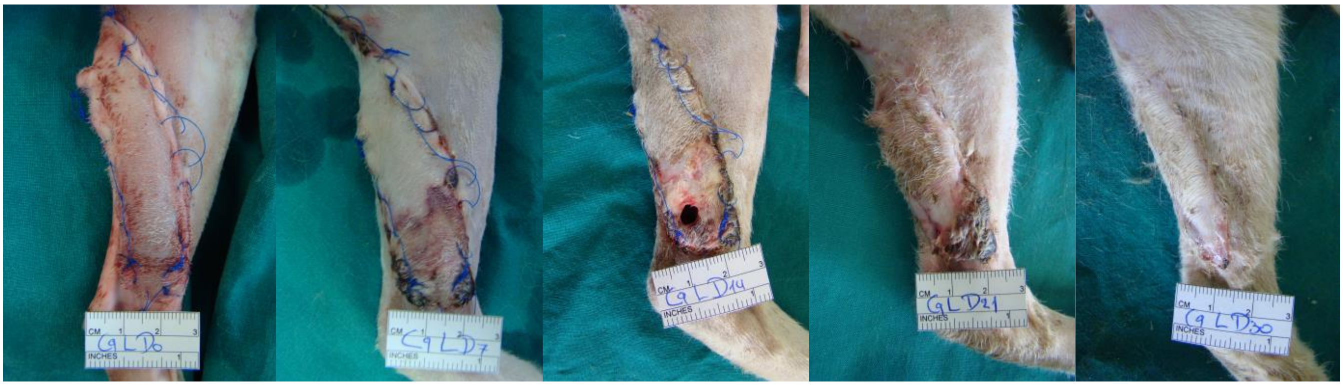

(a) (b) (c) (d) (e)

Figure S1. The healing process of a ST flap (group A) on days 0 (a), 7 (b), 14 (c), 21 (d) and 30 (e) postoperatively

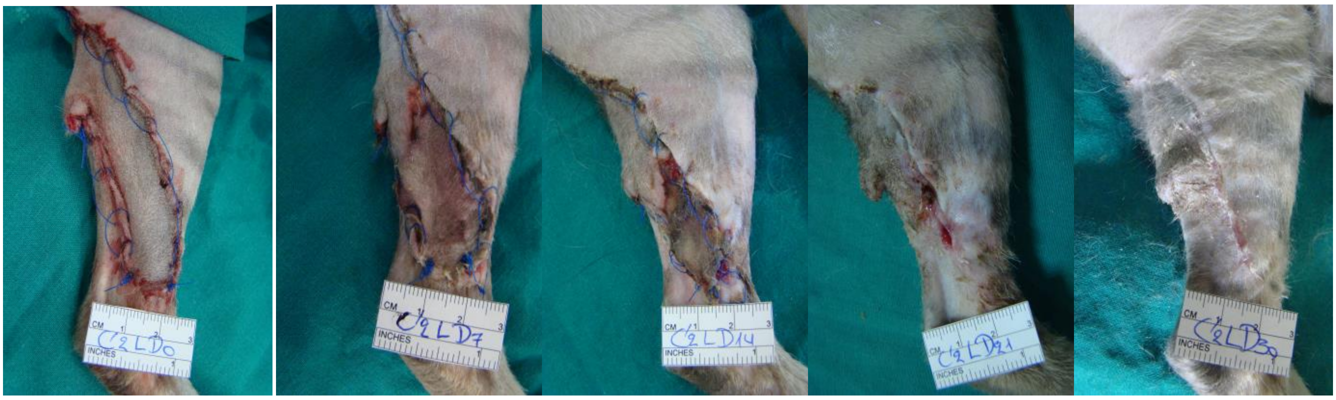

(a) (b) (c) (d) (e)

Figure S2. The healing process of a SST flap (group B) on days 0 (a), 7 (b), 14 (c), 21 (d) and 30 (e) postoperatively

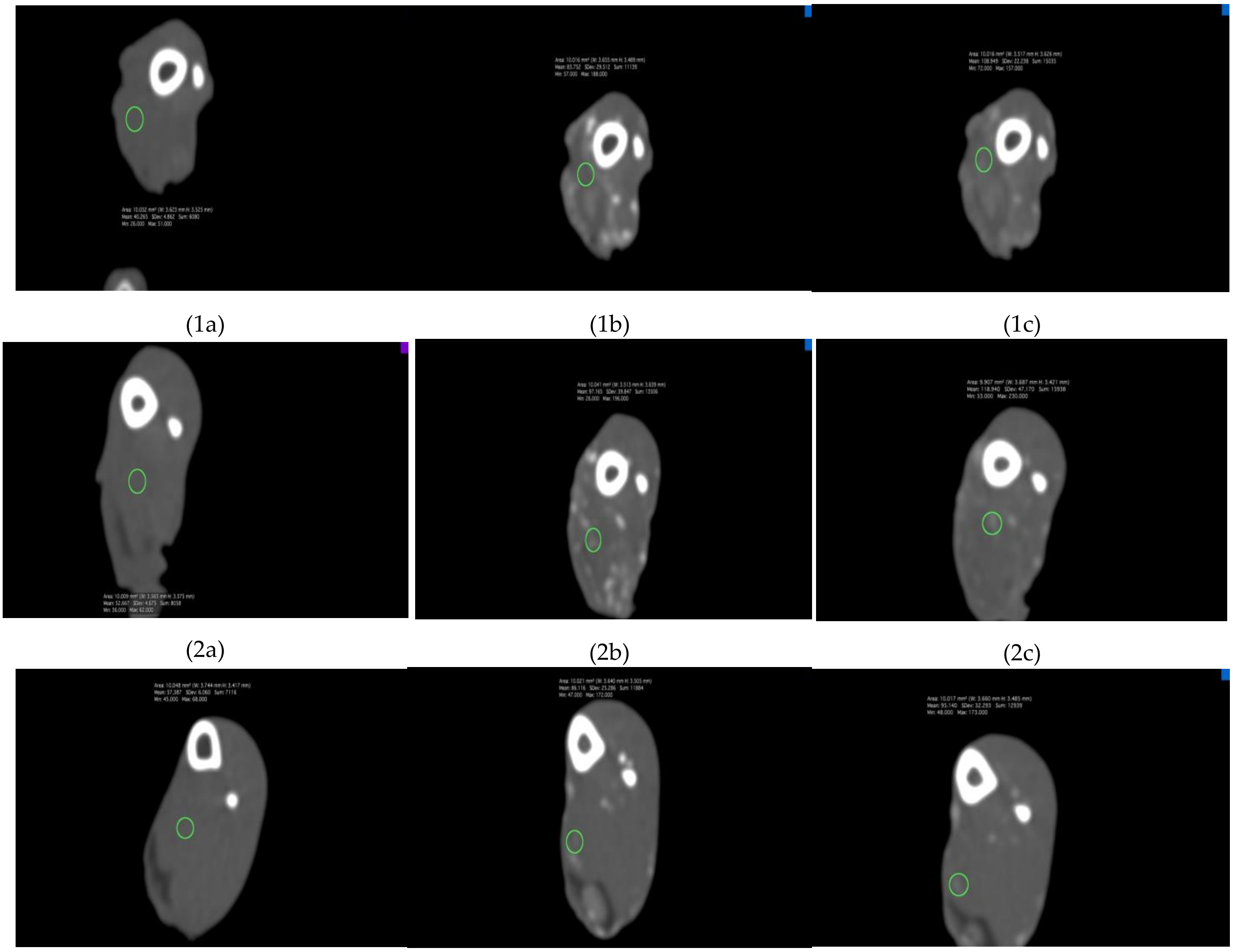

Figure S3. Evaluation of the density of the semitendinosus muscle (HU at $10mm^2$) in group A, 10 days after ST-flap elevation, at three different points [proximal (A), medial (B) and distal (C)] in three different phases [pre-contrast agent (1), arterial phase (2) and venous phase (3)].

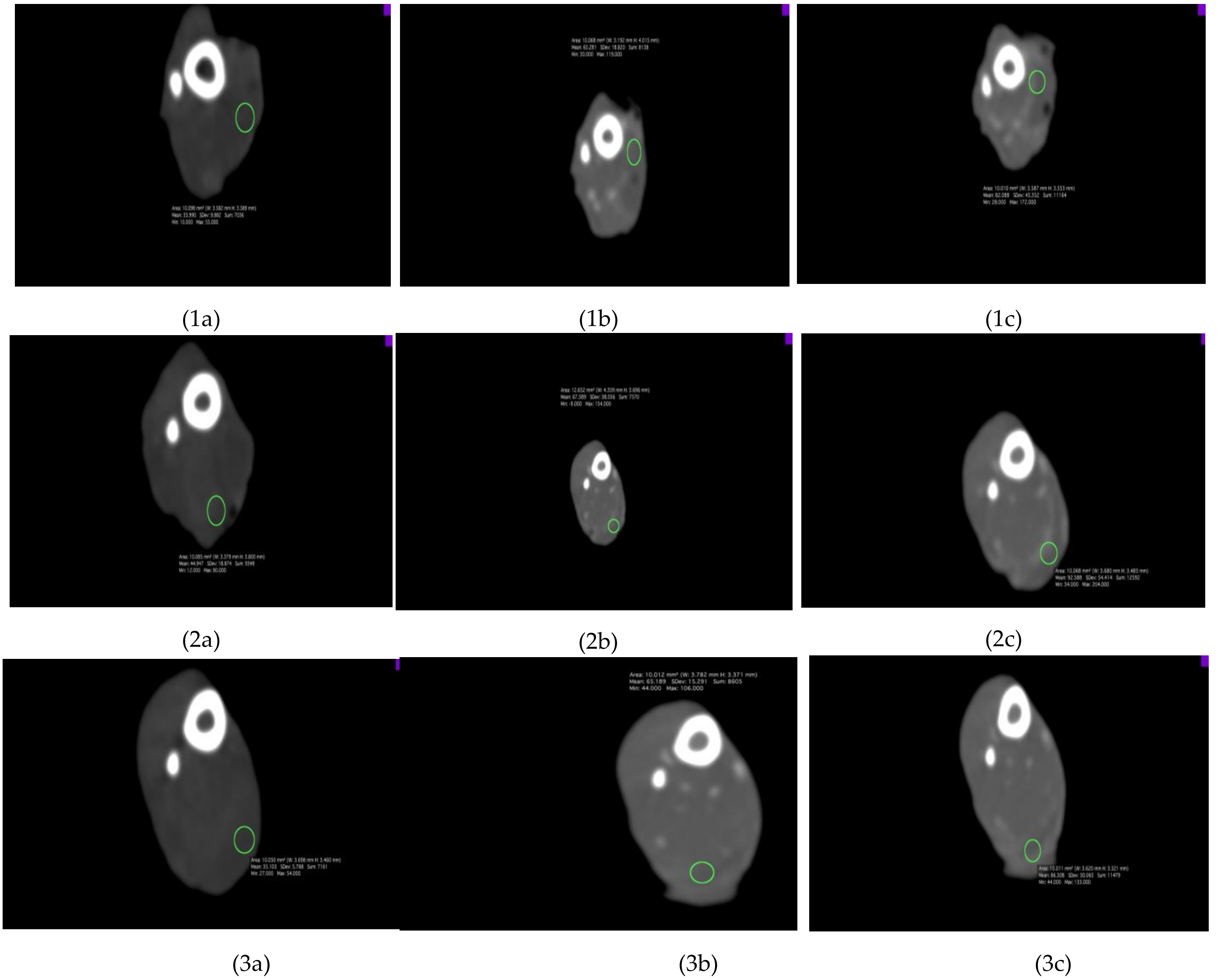

Figure S4. Evaluation of the density of the semitendinosus muscle (HU at 10 mm^2) in group B, 10 days after SST-flap elevation, at three different points [proximal (A), medial (B) and distal (C)] in three different phases [pre-contrast agent (1), arterial phase (2) and venous phase (3)].
